# Direct, but not indirect, predation‐risk cues deter larval settlement in a scyphozoan jellyfish

**DOI:** 10.1111/1365-2656.70357

**Published:** 2026-09-25

**Authors:** Lucas H. Gimenez, Anthony R. Carroll, Kylie A. Pitt

**Affiliations:** ^1^ School of Environment and Science Griffith University Southport QLD Australia

**Keywords:** benthic environment, chemical ecology, habitat selection, invasive species, larval behaviour, predator–prey interaction, recruitment

## Abstract

1. Settlement represents a key transition from dispersal to recruitment, during which predation can strongly influence population dynamics through both consumptive and non‐consumptive effects. Predation generates chemical cues that convey information about the presence of predators, attacks on prey, ingestion of prey and post‐attack conditions. Predation‐risk cues can be direct, when emitted by the predator itself, or indirect, when released by prey, yet how larvae respond to these cues during settlement remains poorly understood in many systems.
2. Here, we assessed how larvae of the scyphozoan jellyfish *Aurelia coerulea* respond to different predation‐risk cues by conducting four experiments manipulating predator kairomones, conspecific alarm cues, conspecific post‐attack cues and predator diet cues. We hypothesised that settlement responses would depend on the information provided by cues, with an avoidance response expected for those indicating direct predation risk.
3. Settlement was deterred in response to direct cues (predator kairomones) and the combination of direct and indirect cues (predator kairomones with conspecific alarm cues). In contrast, settlement was unaffected by indirect cues alone (conspecific post‐attack cues) and cues not associated with predation (mechanical damage cues). In addition, predator diet cues had no further effect beyond that of predator kairomones alone. These results suggest that larval behaviour is shaped by predation risk, with information about the presence of predators playing a key role in eliciting antipredator responses during settlement.
4. Our findings provide mechanistic evidence for understanding how predation‐risk cues mediate non‐consumptive effects during habitat selection in organisms with complex life cycles, highlighting how antipredator responses during a critical life‐history transition can influence recruitment success.

## Graphical abstract

Predators can shape population dynamics before consumption occurs, with prey using chemical information to guide larval settlement. We show that jellyfish larvae (*Aurelia coerulea*) respond selectively to predation‐risk cues, reducing settlement in response to predator presence and active predation cues, but not to residual cues from previously preyed conspecific polyps. These findings highlight how non‐consumptive effects can influence recruitment in organisms with complex life cycles.

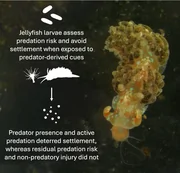

## 1 INTRODUCTION

Predation is a major driver of benthic communities through direct consumptive effects that reduce prey abundance (Oricchio et al., 2016; Winkelmann et al., 2011). Predators also generate non‐consumptive effects when prey detect risk and modify their actions or traits without being attacked (Scrosati, 2021). These antipredator responses can involve changes in behaviour (Dunn et al., 2018), morphology (Solas et al., 2015), chemistry (Wu et al., 2020) or life history (Sniegula et al., 2025). Because such responses are energetically costly, their expression is typically graded and context‐dependent (Chivers et al., 2014; Weissburg et al., 2014). Such non‐consumptive effects can operate at early life‐history stages, be carried‐over across subsequent stages (Ellrich, Scrosati, Bertolini, & Molis, 2016; Solas et al., 2015) and, if persistent and intense enough, can even influence population and community dynamics (Catalán et al., 2021; O'Connor et al., 2013; Sheriff et al., 2020).

The mechanisms that prey rely on to detect information about predators are central to understanding non‐consumptive effects. Prey are exposed to multiple sources of information about predation risk that can differ in immediacy and reliability, including chemical, visual and mechanical cues (Jones et al., 2024; McCormick & Manassa, 2008). As a result, responses may vary depending on the cues encountered, reflecting differences in the information contained in those cues (Ferrari et al., 2010; Wisenden, 2015), yet how this variation shapes antipredator behaviour remains poorly resolved in many systems.

Predator recognition arises through both innate mechanisms shaped by coevolution and learned associations formed through experience (Lönnstedt et al., 2012; Mezrai et al., 2020). Innate responses allow naïve prey to respond immediately to predators but are often restricted to historically encountered threats (Hawkins et al., 2004). When prey face novel or rarely encountered predators, learning enables them to associate cues from predators with risk and respond appropriately (Wisenden, 2015). In biological invasions, where prey must rapidly respond to novel predators, non‐native species often lack innate recognition, making the ability to learn and use available cues critical for determining the nature and strength of behavioural responses (Bible et al., 2017; Castorani & Hovel, 2016).

Chemical cues play key roles in predator–prey interactions (Ferrari et al., 2010). Predation occurs as a sequence of stages including detection, attack, capture and ingestion, each of which can generate distinct cues about risk (Wisenden, 2015). These cues can be classified as direct, when emitted by the predator itself, or indirect, when released by prey or the environment in response to predation (Jones et al., 2024). Kairomones are metabolites in odours or excretions that prey use to detect the presence of predators before an attack (Kamio & Derby, 2017; Wisenden, 2015). During attack and capture, injured prey release alarm cues that indicate active predation (Morishita & Barreto, 2011). These alarm cues are a specific type of damage cues, but not all cues released upon damage provide information about predation risk because injuries can also occur through non‐predatory sources. Alarm cues are thus more effective when paired with predator kairomones (Bourdeau, 2010; Cheung et al., 2006). After ingestion, alarm cues can survive digestion and be re‐emitted by the predator itself as diet cues that signal recent consumption (Mitchell et al., 2015). Prey may also emit post‐attack cues induced by stress from predation pressure that can persist after the predator has left the area, reflecting recent history of predation (Ng & Gaylord, 2020). Together, these cues allow prey to assess predation risk and adjust their responses accordingly to the information available.

Habitat selection is a fundamental behavioural response to predation risk, allowing prey to reduce exposure to predators by choosing where to establish, forage or shelter (Beermann et al., 2018; Boehme‐Terrana et al., 2022). For many sessile organisms, habitat selection occurs during a dispersive larval phase transported by currents, during which individuals make a single, effectively irreversible choice during settlement (Jorissen & Nugues, 2021; Speare et al., 2023). In these populations, recruitment is often constrained by high levels of predation mortality during both the larval phase (Allen, 2008; Fulford et al., 2011) and the early post‐settlement period (Wells et al., 2024). Predation risk can thus directly affect small‐scale settlement choices, a plastic behavioural response to the presence of predators in the area that ultimately enhances recruitment success (Cowan et al., 2016; von der Meden et al., 2015).

Larvae rely on information about habitat quality when selecting a substrate by detecting chemical cues associated with suitable habitats (Bilodeau & Hay, 2022; Hadfield & Paul, 2001). These cues may originate from multiple biological sources, including signals from conspecifics that promote gregarious settlement (Elbourne & Clare, 2010; Zhao & Qian, 2002), as well as predator kairomones and alarm cues from injured conspecifics that indicate elevated predation risk (Ellrich, Scrosati, Romoth, & Molis, 2016; Pruett & Weissburg, 2019). Settlement responses can therefore reflect a trade‐off between habitat suitability and predation risk and may vary depending on the available cues, requiring larvae to respond to potentially conflicting information (Bilodeau & Hay, 2022; Miner et al., 2010).

In scyphozoan jellyfish, free‐swimming larvae attach to suitable substrate and metamorphose into sessile polyps (Helm, 2018; Lucas, 2001). Settlement and recruitment are strongly influenced by abiotic factors such as temperature, salinity and light, as well as chemical cues from microbial biofilms and conspecifics (Dror & Angel, 2025; Gambill et al., 2018; Gimenez et al., 2025a; Holst & Jarms, 2007; Ohdera et al., 2023). Polyp aggregations can persist for years through asexual reproduction and periodically release juvenile medusae, thereby representing a key stage in the life cycle (Helm, 2018; Lucas et al., 2012). Polyp abundance, together with production and survival of juvenile medusae, largely determines the scale and timing of jellyfish blooms (Fernández‐Alías et al., 2024). Despite the importance of settlement for population dynamics, the role of predation risk in shaping larval behaviour has received little attention in this group.

Predation by nudibranchs represents an important top‐down control on jellyfish polyps (Feng et al., 2017). Some species prefer jellyfish polyps over alternatives such as sea anemones (Hoover et al., 2012), whereas others can subsist exclusively on jellyfish polyps for extended periods, particularly when alternative prey is scarce (Dror et al., 2025). Although predation rates vary among species of nudibranchs (Takao et al., 2014; Tang et al., 2021), they exert clear consumptive effects by substantially reducing polyp densities and fragmenting aggregations (Gröndahl, 1988; Hernroth & Gröndahl, 1985; Keen, 1991). This predator–prey system thus provides an opportunity to examine non‐consumptive effects of predators on settlement of scyphozoan jellyfish.

Here, we focused on the jellyfish *Aurelia coerulea* von Lendenfeld, 1884 and the nudibranch *Austraeolis ornata* (Angas, 1864). Originally from East Asia, the jellyfish *A. coerulea* has been introduced to North America, Europe and Oceania, where polyps can form dense aggregations in human‐modified habitats (Gittenberger et al., 2023; Marques et al., 2015, 2019; Scorrano et al., 2016). In southern Australia, the nudibranch *A. ornata* is a voracious native predator of cnidarians that readily consumes non‐native *A. coerulea* polyps, potentially representing a top‐down control in areas where their distributions overlap (Gimenez et al., 2025b). Using this system, we tested whether larval behaviour varies among chemical cues generated by predation. These cues typically occur simultaneously and form a dynamic landscape of information. To isolate their effects, we conducted four experiments to quantify settlement in response to: (1) predator kairomones; (2) conspecific alarm cues; (3) conspecific post‐attack cues; and (4) predator diet cues. We hypothesised that settlement responses would depend on the information provided by cues, with an avoidance response expected for those indicating direct predation risk.

## 2 MATERIALS AND METHODS

### 2.1 Collection and maintenance of animals

*Aurelia coerulea* polyps and larvae were obtained from cultures at the Sea Jellies Illuminated exhibit, Sea World Australia. Based on genetic analyses, these cultures originate from New South Wales and/or Victoria in southern Australia (P. E. Diaz, pers. comm.), where the jellyfish *A. coerulea* is an introduced species and the native nudibranch *A. ornata* also occurs (Atlas of Living Australia, 2026; iNaturalist, 2026).

Fully developed polyps (~4 mm height, 16 tentacles) were transferred onto 90 mm plastic dishes, with about 200 individuals per dish, and submerged in 15 L aquaria to allow reattachment. They were maintained with gentle aeration and fed *Artemia* sp. weekly for about 8 weeks, by which time they had formed small patches by asexual reproduction (approximately 850 individuals per dish).

Brooding medusae maintained in the same culture system were used to obtain larvae. For each experiment, larvae from six medusae (10–20 cm bell diameter) were pooled to minimise maternal effects. Competent larvae (~300 μm length, elongated shape, actively swimming with direction) were transferred to 1 L of filtered seawater. Larval concentrations (individuals mL^−1^) were quantified from subsamples (*n* = 10).

*Austraeolis ornata* nudibranchs were collected from Burleigh Heads, Australia (28°5′21.0″ S, 153°27′23.3″ E) under Queensland Government permit no. 269483. They were maintained for 24–48 h before the experiments in 100 L recirculating aquaria containing rocks from the sampling location as habitat.

All animals were kept at 18–19°C, 33–34 ppt salinity and a 15/9 h light/dark photoperiod across all experiments to avoid confounding effects. Experiment 1 was conducted in September 2024, and Experiments 2–4 in June–July 2025. No ethical approval was required for this study, and all procedures complied with relevant institutional, national and international guidelines for the care and use of marine organisms.

### 2.2 Experiments

Each experiment targeted distinct cues to assess how settlement responses varied among direct and indirect information about predation risk. Chemical cues were collected immediately before settlement assays by conditioning artificial seawater (Red Sea Salt dissolved in reverse osmosis water) with the relevant organisms for each treatment. Conditioned seawater was prepared in different ways depending on the cues: nudibranchs were used to obtain predator kairomones, polyps exposed to active predation were used to obtain conspecific alarm cues, polyps previously exposed to predation were used to obtain conspecific post‐attack cues, and nudibranchs maintained on specific diets were used to obtain predator diet cues. In addition, polyps were mechanically abraded to produce damage cues from non‐predatory injuries. For each experiment, replicates were randomly assigned to treatments and run simultaneously to avoid temporal bias. Replicates were prepared independently, including the conditioning period, and spatially interspersed to minimise position effects.

#### 2.2.1 Experiment 1: Predator kairomones

Larval response to kairomones released by predators was assessed with three treatments: control, predator presence and predator odour. The control treatment contained unconditioned seawater. The predator presence treatment included a live nudibranch that released chemical cues to the water and could prey on newly recruited polyps. The predator odour treatment included seawater that had been conditioned with a live nudibranch (concentration = 1 individual L^−1^, duration = 4 days). Nudibranchs were randomly assigned to treatments and used once. The mean body lengths of nudibranchs did not differ between treatments (overall mean ± SD = 21.8 ± 2.7 mm; Independent *t*‐test, t12 = 0.48, *p* = 0.64).

Replicates (*n* = 7 per treatment; Figure S1) consisted of 1 L aquaria (length × width × height = 21 × 15 × 6 cm) that were gently aerated and contained one inverted dish (diameter = 90 mm) with grooves for larval attachment on the underside (Holst & Jarms, 2007; Pitt, 2000). One hundred larvae were added to each replicate, and the numbers that settled (that had attached and were undergoing or completed metamorphosis) were counted after 72 h. We hypothesised that fewer larvae would settle in both the predator (consumptive and non‐consumptive effects) and odour treatments (non‐consumptive effect).

#### 2.2.2 Experiment 2: Conspecific alarm cues

The response of larvae to alarm cues released by conspecific polyps being attacked by predators was assessed with four treatments: control, uninjured conspecifics, conspecifics injured by predator attack and conspecifics injured via mechanical abrasion. The control treatment contained unconditioned seawater. The other treatments contained seawater that had been conditioned with patches of polyps (concentration = ~850 individuals L^−1^, duration = 1 week). Uninjured conspecifics were used to account for the attraction effect exerted by polyps during gregarious settlement (Gimenez et al., 2025a). Conspecifics injured by predator attack were obtained by allowing a nudibranch to prey on patches of polyps for four 1‐h events over 1 week. Each replicate contained a randomly assigned nudibranch of similar body length (mean ± SD = 25.9 ± 2.4 mm), such that each polyp patch interacted with only a single predator. After each predation event, nudibranchs were allowed to feed on other polyps to ensure satiation and were then starved for at least 24 h before the next event to standardise hunger. Because predators were present during conditioning, this treatment generated repeated pulses of conspecific alarm cues associated with predator kairomones. Conspecifics injured by mechanical abrasion sustained non‐predatory injuries inflicted with a fine needle, which was used to tear the oral disc, tentacles and upper area of the stalk of polyps, in four 1‐h events over 1 week. The number of polyps mechanically injured was consistent with the feeding rates of *A. ornata* observed during predation attacks (7–9 polyps every 15 min).

Replicates (*n* = 7 per treatment; Figure S2) consisted of 400 mL beakers without aeration and one inverted dish (diameter = 52 mm) with grooves for larval attachment. Sixty larvae were added per replicate and the numbers that settled were counted after 72 h. We hypothesised that settlement would be lowest in the predator‐injured treatment (alarm cues and predator kairomones), intermediate in the mechanically injured treatment (non‐predatory damage cues) and highest in the uninjured treatment (gregarious settlement cues).

#### 2.2.3 Experiment 3: Conspecific post‐attack cues

Larval response to post‐attack cues from conspecific polyp patches exposed to predation pressure was assessed with three treatments: control, non‐preyed conspecifics and preyed conspecifics. The control treatment contained unconditioned seawater. The other two treatments contained seawater that had been conditioned with patches of polyps. Non‐preyed conspecifics were not exposed to predators, while preyed conspecifics were injured by predation attacks. Predation events followed Experiment 2 but lasted for 3 weeks, with four 1‐h predation events every week, to generate patches with a history of recent persistent predation.

Following the final predation event, nudibranchs were removed and polyp density was standardised to ~400 polyps by carefully detaching excess polyps from each replicate in both treatments. A fine needle was used to detach polyps at the pedal disc in a single swift movement, taking care to avoid damage to the oral disc, tentacles or stalk. Polyp patches were left overnight to recover from handling and the seawater was then fully exchanged to remove any remaining conspecific alarm cues and predator kairomones. This seawater exchange was also applied to the non‐preyed treatment to standardise conditions across treatments. The subsequent conditioning period (concentration = ~400 individuals L^−1^, duration = 1 week) allowed potential conspecific post‐attack cues to accumulate from polyps in patches that had experienced predation. These cues may include stress‐related metabolites, altered mucus composition and other physiological or metabolic changes that persist after predation.

Replicates were established as per Experiment 2. We hypothesised that settlement would be lower in the preyed conspecifics treatment (post‐attack cues) than in the non‐preyed conspecifics treatment (gregarious settlement cues).

#### 2.2.4 Experiment 4: Predator diet cues

The response of larvae to diet cues released after predators had consumed *A. coerulea* polyps was assessed with three treatments: control, predators fed *A. coerulea* polyps and predators fed other prey. The sea anemone *Actinia tenebrosa* was used as the alternative diet because it is a confirmed prey of *A. ornata* nudibranchs (Gimenez et al., 2025b).

The control treatment had unconditioned seawater. Each diet treatment included conditioned seawater with nudibranchs previously fed exclusively on *A. coerulea* polyps or *A. tenebrosa* sea anemones for 3 weeks. This design allowed the separation of general predator kairomones from potential diet cues associated with recent consumption of *A. coerulea* polyps. For each replicate, a nudibranch was randomly assigned to a treatment and used once, and mean body length of nudibranchs did not differ between treatments (overall mean ± SD = 25.5 ± 2.2 mm; Independent *t*‐test, *t*_12_ = 0.78, *p* = 0.44). Prey was provided ad libitum, with new jellyfish polyps or sea anemones added daily after checking availability. After 3 weeks, any remaining prey was removed and the seawater was fully exchanged, ensuring that only cues from nudibranchs accumulated during conditioning (concentration = 1 nudibranch L^−1^, duration = 4 days).

Replicates were established as per Experiment 2. We hypothesised that settlement would be lower in the predators fed *A. coerulea* polyps treatment (predator diet cues) than in the predators fed *A. tenebrosa* sea anemones treatment (general predator kairomones).

### 2.3 Data analysis

Settlement was analysed using Generalised Linear Models (GLMs) with a binomial distribution and logit link. The response was specified as the number of settled larvae (successes) out of the total number of larvae added (trials), consistent with a binomial sampling process (Warton & Hui, 2011). Overdispersion was checked via Pearson's chi‐square ratio, and when present, models were refitted using a quasibinomial distribution to adjust standard errors (Zuur et al., 2009). Model adequacy was verified by inspecting residual patterns. A summary of the model structure for each experiment, along with the overall equation of the model, is provided in Table S1.

Control treatments were used as the reference level, and pairwise comparisons of estimated marginal means were conducted with Tukey adjustment for multiple testing. Analyses were done in R version 4.4.3 (R Core Team, 2025). Estimated marginal means and Tukey‐adjusted pairwise comparisons were obtained using the *emmeans* package (Lenth et al., 2025). Compact letter displays were generated using the *multcompView* package (Graves et al., 2026). Figures present observed settlement percentages (mean ± SD), while statistical comparisons are based on odds ratios from the models.

## 3 RESULTS

Model outputs assessing treatment effects on settlement in each experiment are provided in Table S2 and pairwise comparisons of estimated marginal means for settlement among treatments are summarised in Table S3.

### 3.1 Experiment 1: Predator kairomones

Settlement was highest in the control (26.0 ± 6.5%), lower in seawater conditioned with predator odour (16.6 ± 5.7%) and lowest in the presence of predators (8.7 ± 6.2%; Figure 1). Compared with the control, settlement odds were lower in the presence of live nudibranchs (OR = 0.27, *p* < 0.001) and in seawater conditioned with nudibranch odour (OR = 0.56, *p* = 0.041). Settlement odds were also lower in the predator presence treatment than the predator odour treatment (OR = 0.48, *p* = 0.037).

**FIGURE 1:**
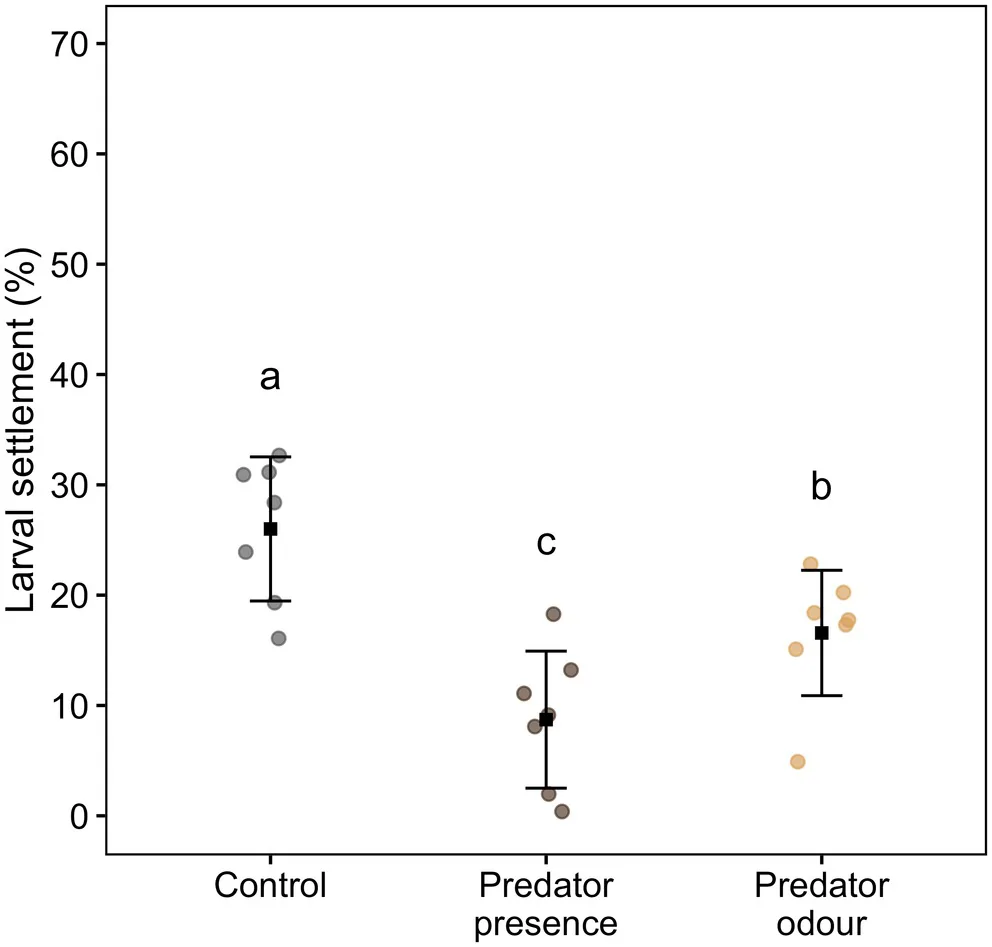
Settlement of *Aurelia coerulea* larvae exposed to predators and their cues. Data are mean ± standard deviation, along with individual values. Treatments: Control (no predator effect), predator presence (combined consumptive and non‐consumptive effects) and predator odour (non‐consumptive effect). Treatments with different letters show significant differences in means (*p* < 0.05).

### 3.2 Experiment 2: Conspecific alarm cues

Settlement was higher in seawater conditioned with mechanically abraded conspecifics (43.3 ± 10.7%) and uninjured conspecifics (41.2 ± 9.9%) than in the control (28.1 ± 6.6%), whereas settlement was lower in seawater conditioned with predator‐attacked conspecifics (16.4 ± 5.3%; Figure 2). Compared with the control, settlement odds were higher with cues from mechanical abrasion (OR = 1.96, *p* = 0.006) and uninjured conspecifics (OR = 1.79, *p* = 0.023), but lower with cues from predator attack (OR = 0.50, *p* = 0.020). Settlement odds were also lower with cues from predator attack than with cues from mechanical abrasion (OR = 0.26, *p* < 0.001) and uninjured conspecifics (OR = 0.28, *p* < 0.001). The mechanical abrasion and uninjured conspecifics treatments did not differ from each other (*p* = 0.969).

**FIGURE 2:**
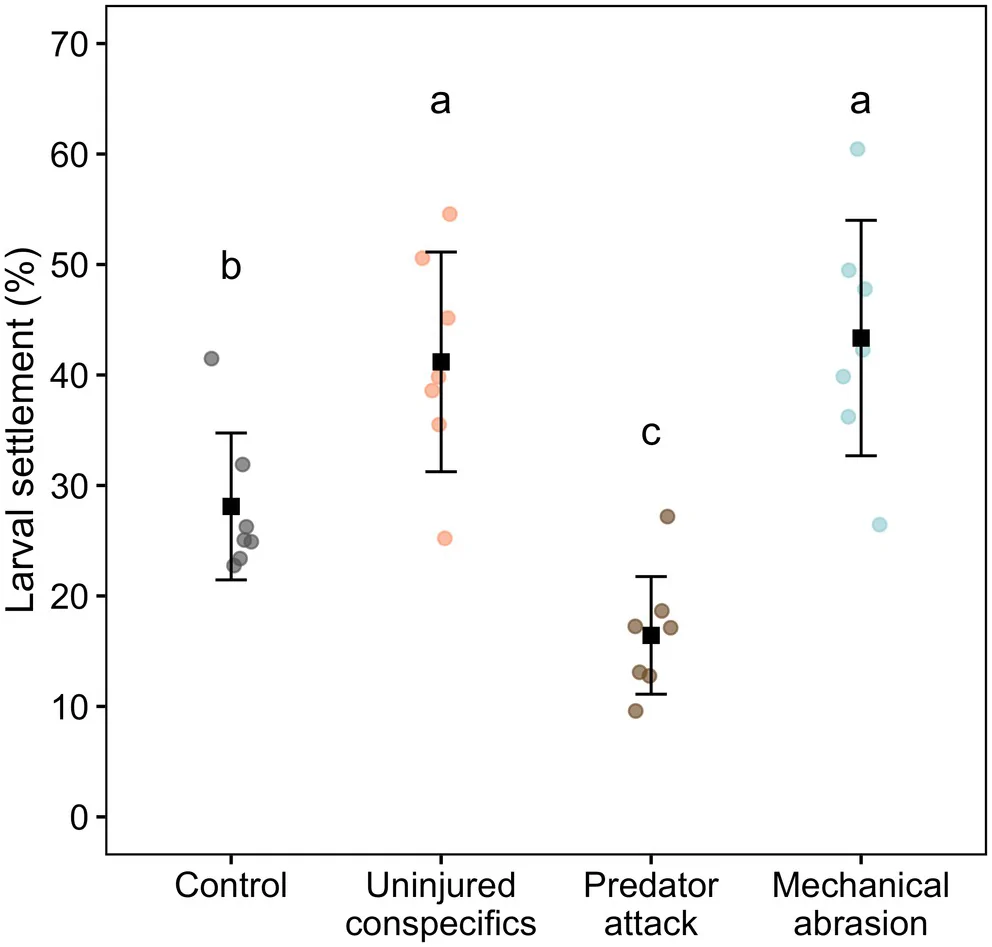
Settlement of *Aurelia coerulea* larvae in response to chemical cues from conspecifics under different predation scenarios. Data are mean ± standard deviation, along with individual values. Treatments: Control (no cues), uninjured conspecifics (gregarious settlement cues), conspecifics injured by predator attack (alarm cues and predator kairomones) and conspecifics injured by mechanical abrasion (non‐predatory damage cues). Treatments with different letters show significant differences in means (*p* < 0.05).

### 3.3 Experiment 3: Conspecific post‐attack cues

Settlement was higher in seawater conditioned with non‐preyed conspecifics (38.6 ± 7.6%) and preyed conspecifics (35.7 ± 7.7%) than in the control (25.2 ± 8.5%; Figure 3). Compared with the control, settlement odds were higher with cues from non‐preyed conspecifics (OR = 1.86, *p* = 0.006) and preyed conspecifics (OR = 1.65, *p* = 0.036). The non‐preyed and preyed conspecifics treatments did not differ from each other (*p* = 0.796).

**FIGURE 3:**
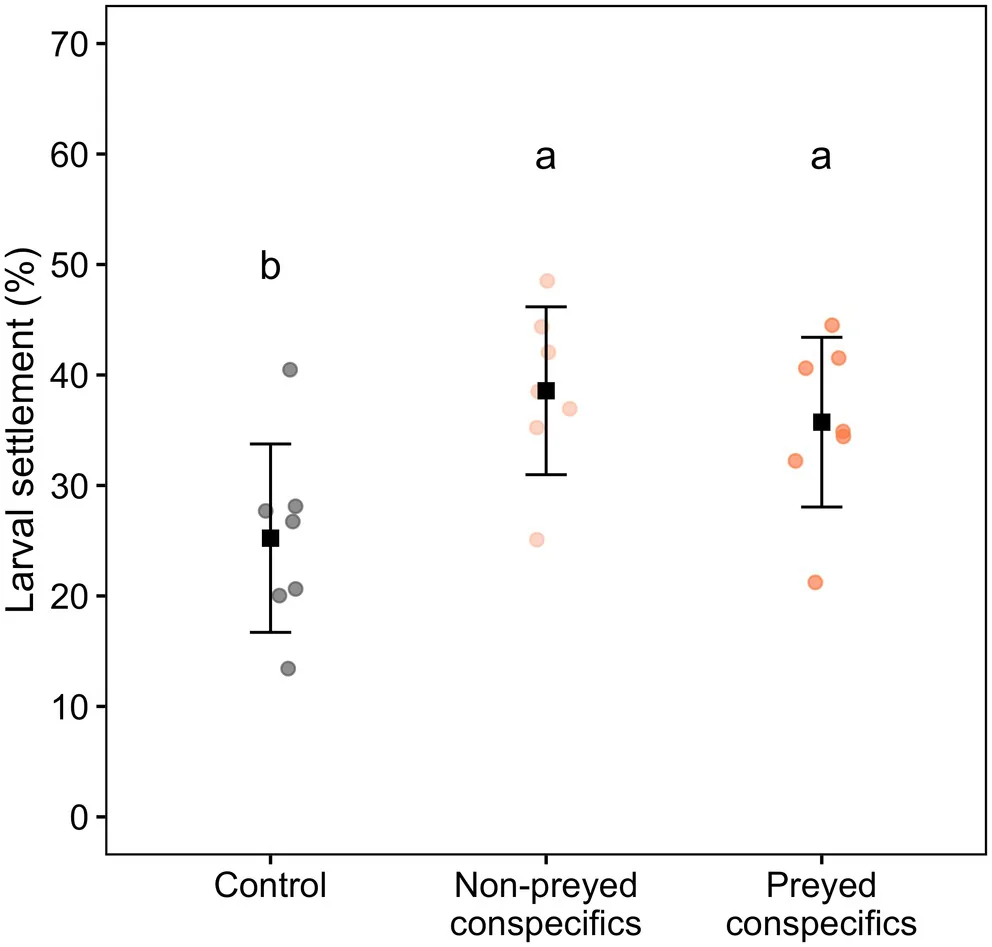
Settlement of *Aurelia coerulea* larvae in response to chemical cues from conspecifics subjected to different short‐term predation pressure. Data are mean ± standard deviation, along with individual values. Treatments: Control (no cues), non‐preyed conspecifics (gregarious settlement cues) and preyed conspecifics (post‐attack cues). Treatments with different letters show significant differences in means (*p* < 0.05).

### 3.4 Experiment 4: Predator diet cues

Settlement was higher in the control (27.9 ± 6.5%) than in seawater conditioned with predators fed sea anemones (20.5 ± 6.3%) and jellyfish polyps (16.4 ± 6.1%; Figure 4). Compared with the control, settlement odds were lower with cues from nudibranchs fed *A. tenebrosa* sea anemones (OR = 0.67, *p* = 0.034) and *A. coerulea* polyps (OR = 0.51, *p* < 0.001). The two diet treatments did not differ from each other (*p* = 0.296).

**FIGURE 4:**
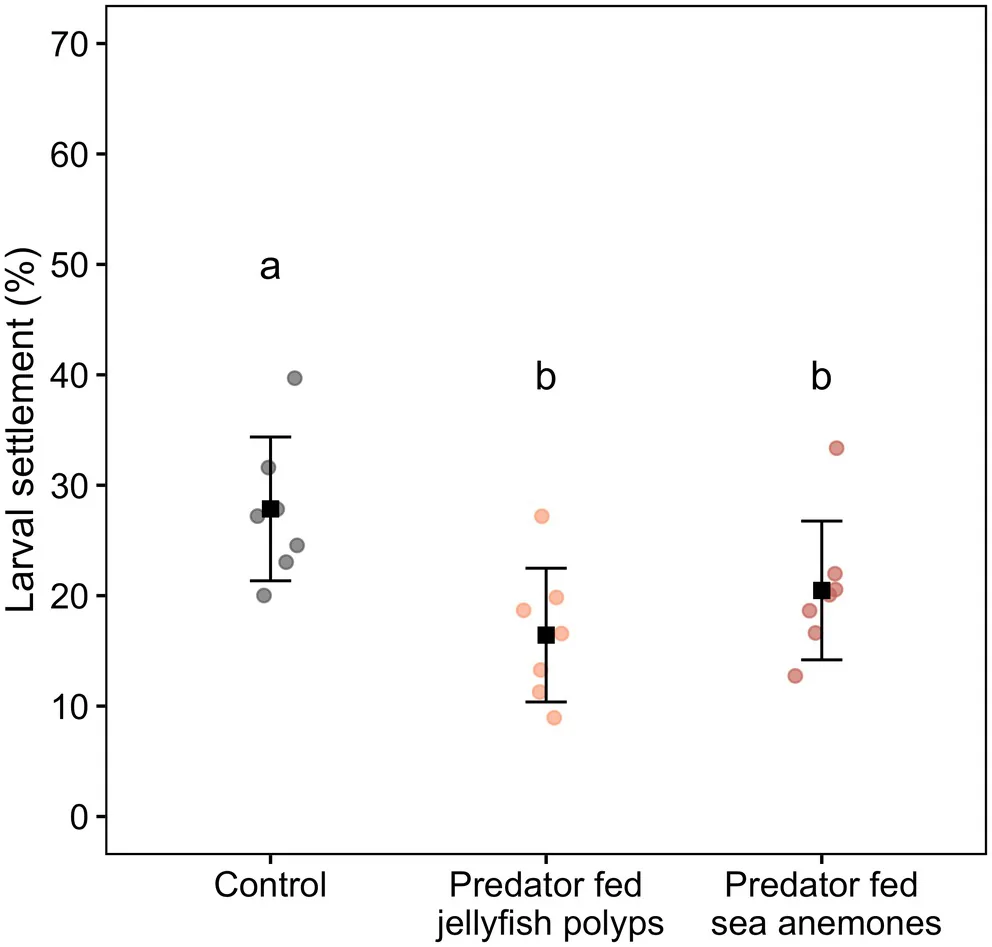
Settlement of *Aurelia coerulea* larvae in response to chemical cues from predators maintained on different diets. Data are mean ± standard deviation, along with individual values. Treatments: Control (no cues), predator fed *A. coerulea* jellyfish polyps (predator diet cues) and predator fed *A. tenebrosa* sea anemones (general predator kairomones). Treatments with different letters show significant differences in means (*p* < 0.05).

## 4 DISCUSSION

Our study provides evidence that larval behaviour in *A. coerulea* is shaped by predation risk, with settlement responses determined by whether predation‐risk cues provided direct or indirect information. Larvae responded to predator kairomones released by nudibranchs that signal their presence, and to the combination of these kairomones with alarm cues emitted by conspecific polyps that signal predation attacks. In contrast, larvae did not show evidence of responding to conspecific post‐attack cues that signal residual predation risk, nor to predator diet cues that signal recent consumption of specific prey. Moreover, responses to damage cues differed between predatory and non‐predatory injuries, indicating that injury alone was insufficient to elicit an antipredator response. Together, these results show a consistent pattern: direct predation‐risk cues reduced settlement, whereas indirect predation‐risk cues did not have a detectable effect when direct cues were absent.

A key challenge in studies of chemical cues mediating predation risk assessment is that the effects of different cues are often difficult to disentangle. Predator kairomones, conspecific alarm cues, conspecific post‐attack cues and predator diet cues differ in the information they contain, which influences how risk is perceived (McCoy et al., 2012; Mitchell et al., 2017). These cues occur simultaneously and can vary greatly in their persistence and range, creating temporally and spatially dynamic landscapes of information (Van Buskirk et al., 2014; Weissburg et al., 2014). By isolating distinct predation‐risk cues, our results clarify how each of them influences settlement.

Settlement of *A. coerulea* larvae was reduced when exposed to either live nudibranchs or their odour, with a stronger effect observed when predators had access to recently settled recruits (Experiment 1). This pattern likely arises from the simultaneous action of direct predation and chemically mediated avoidance: nudibranchs remove newly settled polyps while also releasing kairomones that signal risk to approaching larvae. Nudibranchs are known to exert strong top‐down control on jellyfish polyp populations (Gröndahl, 1988; Hernroth & Gröndahl, 1985; Keen, 1991), primarily through high consumption rates (Dror et al., 2025; Takao et al., 2014; Tang et al., 2021). In particular, *A. ornata* can consume up to ~200 fully developed *A. coerulea* polyps day^−1^ (Gimenez et al., 2025b), with potentially even higher rates on small, recently settled polyps. Such consumption of new recruits may also release conspecific alarm cues that, combined with predator kairomones, may elicit a stronger antipredator response than kairomones from nudibranchs alone.

Beyond direct predation, live nudibranchs act as persistent sources of kairomones, maintaining stronger and more stable chemical signalling than conditioned seawater, where compounds likely degrade over time. The fact that seawater conditioned with nudibranch odour still reduced settlement indicates that larvae respond to predator kairomones during habitat selection. Exposure to such predator cues is therefore likely to delay settlement, with larvae remaining in the water column where passive transport by local hydrodynamics may move them away from high‐risk areas. Such non‐consumptive effects can drive settlement patterns and determine recruitment success, reinforcing the role of nudibranch predators on the population dynamics of cnidarian prey. Similar predator‐induced settlement deterrence has been documented in other sessile invertebrates with complex life cycles, including barnacles (Ellrich, Scrosati, Bertolini, & Molis, 2016; Yorisue et al., 2019), mussels (Ehlers et al., 2018) and oysters (Pruett & Weissburg, 2019).

Chemical cues from active predation on conspecific polyps also reduced settlement (Experiment 2). Because the predator attack inherently involves both conspecific alarm cues and predator kairomones, the relative contribution of each cannot be fully disentangled. Prey rely on this association to evaluate predation risk and elicit selective responses (James & McClintock, 2017; Kenison et al., 2018; Mitchell & McCormick, 2013; Pruett & Weissburg, 2019). Evidence consistently shows that conspecific alarm cues are more effective when paired with predator kairomones because each provides complementary information: alarm cues indicate that conspecifics are being attacked, while kairomones identify the predator responsible (Wisenden, 2015).

Mechanical abrasion on conspecific polyps did not reduce settlement, suggesting that non‐predatory damage cues were offset by the positive effect on settlement associated with the presence of conspecifics (Gimenez et al., 2025a). Because polyps were injured at a similar rate in the mechanical abrasion and predator attack treatments, any effects of reduced polyp density are unlikely to explain the contrasting outcomes. Although alarm cues originate from injured conspecifics, they can be functionally distinct from the broader class of damage‐released cues because the former reliably signal predation risk and elicit antipredator responses, whereas the latter may not convey any information about predation risk (Ferrari et al., 2010; Schoeppner & Relyea, 2005; Wisenden, 2000). Alternatively, injuries may release equivalent cues regardless of source (non‐predatory or predatory), but only signal predation risk when paired with additional information indicating the presence of a predator. Similar patterns have been reported in oyster larvae, where cues from injured conspecifics may influence larval responses but do not always produce a consistent reduction in settlement when presented alone (Pruett & Weissburg, 2019).

The association between conspecific alarm cues and predator kairomones can facilitate predator recognition (Larson & McCormick, 2005). This associative learning process is particularly relevant in biological invasions, where novel predator–prey interactions arise (Grason & Miner, 2012). In cnidarians, both non‐associative and associative learning have been reported (Cheng, 2021). Recent work suggests that behavioural responses of *A. coerulea* polyps to cues from prey may be influenced by previous feeding experience (Gregorin et al., 2025), suggesting some degree of behavioural plasticity. The observed reduction in settlement in response to seawater conditioned with *A. ornata* nudibranchs shows that *A. coerulea* larvae detect cues from predators when selecting a substrate, even in the absence of injured conspecifics. Given that both species have been reported in southern Australia for more than a century (Angas, 1864; iNaturalist, 2026; von Lendenfeld, 1884), both evolved and experience‐dependent mechanisms of predator recognition remain plausible.

For many sessile organisms, the presence of conspecifics signals suitable habitats (Prendergast et al., 2008; Sedanza et al., 2022), whereas the presence of predators indicates elevated post‐settlement mortality risk (Cowan et al., 2016; Miner et al., 2010). Settlement is thus influenced by the relative strength of these cues (Morello & Yund, 2016; Sniegula et al., 2025). This trade‐off can be further constrained by time‐dependent declines in larval selectivity as individuals approach the end of their competence period (Marshall & Keough, 2003). In barnacles, attraction to conspecifics can offset predator deterrence (Ellrich, Scrosati, Romoth, & Molis, 2016). By contrast, our results suggest that predator cues reduced *A. coerulea* settlement even in the presence of conspecific cues. Such differing outcomes can be shaped by prey and predator densities (Boudreau et al., 2018; Ellrich, Scrosati, & Molis, 2015; Ellrich, Scrosati, & Petzold, 2015; Hill & Weissburg, 2013) and local hydrodynamics (Ellrich & Scrosati, 2016; Kimbro et al., 2020). Jellyfish polyps form dense aggregations comprising hundreds of thousands of individuals (Marques et al., 2019; Willcox et al., 2008), whereas nudibranchs can aggregate around food (Otter et al., 2025). Such spatially clustered distributions may generate localised cue intensity, affecting larval selectivity at fine spatial scales.

Recent predation history did not affect settlement in the absence of predators (Experiment 3). Prey responses to predators are not always limited to their instantaneous presence, as individuals previously exposed to predators can retain the capacity to elicit antipredator behaviour in conspecifics long after the predator itself has left the area (Ng & Gaylord, 2020). In our experiment, larvae were exposed to cues from polyp patches with contrasting predation history, with conspecific alarm cues and predator kairomones removed, to isolate any lasting chemical signal in remaining polyps (conspecific post‐attack cues) that might signal an unsuitable habitat for settlement. Under these conditions, however, larvae showed a similar response to baseline conspecific cues, indicating that any residual signal of predation risk alone did not modify settlement behaviour, or that it was offset by gregarious settlement cues.

Prey responses can become more pronounced when predators are digesting the same prey species compared to alternative prey (Schoeppner & Relyea, 2009; Weissburg et al., 2016; Weissburg & Beauvais, 2015). In nudibranch‐cnidarian interactions, diet cues can persist in the gut of nudibranchs for several days (Howe & Harris, 1978). Settlement of *A. coerulea* larvae was reduced when exposed to cues from predators regardless of whether they had eaten the same prey species or alternative prey, indicating that larvae responded to predator kairomones and that additional information derived from diet cues did not affect settlement further (Experiment 4). Alternatively, prey may respond to alarm cues from distantly related species that occupy the same ecological guild (Chivers et al., 2002; Mitchell et al., 2012), a pattern also observed in marine invertebrates such as crustaceans and echinoderms (Briones‐Fourzán et al., 2008; Parker & Shulman, 1986).

## 5 CONCLUSION

Settlement in *A. coerulea* was reduced when cues conveyed direct information about predation risk. In contrast, indirect information from conspecifics alone did not elicit an antipredator response when predator cues were absent. The specificity of these responses indicates that not every signal associated with predation will have an effect on larvae. In this system, direct predation‐risk cues were the primary drivers of antipredator behaviour, potentially influencing recruitment success by altering settlement patterns. In many organisms with complex life cycles, where survival in subsequent stages depends on habitat selection during settlement, this behaviour suggests that predators may influence population dynamics well before consumption even occurs. Furthermore, in invaded systems, such non‐consumptive effects might represent an additional mechanism through which native predators influence the establishment or spread of invasive species.

## AUTHOR CONTRIBUTIONS

All authors contributed to the study's conception and design. Lucas H. Gimenez led field trips, carried out laboratory experiments and analysed the data. All authors interpreted the results. Lucas H. Gimenez led the writing of the manuscript. Kylie A. Pitt and Anthony R. Carroll significantly contributed to editing the manuscript and critical review. All authors read and approved the final manuscript.

## FUNDING INFORMATION

This work was funded by Griffith University, which had no role in study design, data collection and analysis, decision to publish or preparation of the manuscript.

## CONFLICT OF INTEREST STATEMENT

The authors declare no conflict of interest.

## Supporting information


**Figure S1.** Schematic of the experimental replicate used in Experiment 1.
**Figure S2.** Schematic of the experimental replicate used in Experiments 2, 3 and 4.
**Table S1.** Summary of model structure for each experiment.
**Table S2.** Summary of each model assessing the effect of treatments on settlement across experiments.
**Table S3.** Pairwise comparisons of estimated marginal means for settlement across treatments.

## Data Availability

Data available from the Dryad Digital Repository: https://doi.org/10.5061/dryad.g79cnp66v (Gimenez et al., 2026).
