# Supplementary material for: Direct, but not indirect, predation‐risk cues deter larval settlement in a scyphozoan jellyfish
Source: J Anim Ecol. 2026 Sep 25;95(11):2335–46. doi: 10.1111/1365-2656.70357 (PMC13731833; doi:10.1111/1365-2656.70357)
Supplement: Supplementary file 1 [file JANE-95-2335-s001.docx]

*Supporting Information*

**Direct, but not indirect, predation-risk cues deter larval settlement in a scyphozoan jellyfish**

Lucas H. Gimenez^*^, Anthony R. Carroll, Kylie A. Pitt

School of Environment and Science, Griffith University, Southport, QLD, Australia

^*^ Corresponding author: [lucas.hernan.gimenez@gmail.com](mailto:lucas.hernan.gimenez@gmail.com)

**Fig. S1.** Schematic of the experimental replicate used in Experiment 1. Each replicate consisted of a 1 L aquarium (length x width x height = 21 x 15 x 6 cm) containing an inverted settlement dish (diameter = 90 mm, grey) floating at the water surface.

**Fig. S2.** Schematic of the experimental replicate used in Experiments 2, 3 and 4. Each replicate consisted of a 400 mL beaker containing an inverted settlement dish (diameter = 52 mm, grey) floating at the water surface.

**Table S1.** Summary of model structure for each experiment. GLM: Generalized Linear Model.

| **Experiment** | **Model** | **Overdispersion** | **Distribution** | **Fixed factor** |
| --- | --- | --- | --- | --- |
| Experiment 1 | GLM | Yes | Quasibinomial | Treatment |
| Experiment 2 | GLM | Yes | Quasibinomial | Treatment |
| Experiment 3 | GLM | Yes | Quasibinomial | Treatment |
| Experiment 4 | GLM | No | Binomial | Treatment |

The overall equation for the models was specified as:

$$Y_{i} \sim Binomial(n_{i},p_{i})$$

$$logit\left( p_{i} \right)= \beta_{0}+\sum_{j=1}^{k} \beta_{j}X_{ji}$$

where *Y_i_* is the number of settled larvae out of *n_i_* larvae added, *p_i_* is the probability of settlement, *β_0_* is the intercept, and *β_j_* are the coefficients for predictor variables *X_j_*.

**Table S2.** Summary of each model assessing the effect of treatments on settlement across experiments. Model estimates are presented on the log-odds scale. Asterisks (*) indicate statistically significant effects (p < 0.05). GLM = Generalized Linear Model; SE = Standard Error.

| **Experiment**  **(model, distribution)** | **Fixed Effect** | **Estimate ± SE** | **t / z-value** | **p-value** |
| --- | --- | --- | --- | --- |
| Experiment 1  (GLM, quasibinomial) | Intercept | −1.046 ± 0.153 | −6.86 | p < 0.001* |
|  | Predator odour | −0.570 ± 0.236 | −2.42 | p = 0.026* |
|  | Predator presence | −1.303 ± 0.282 | −4.62 | p < 0.001* |
| Experiment 2  (GLM, quasibinomial) | Intercept | −0.940 ± 0.151 | −6.21 | p < 0.001* |
|  | Mechanical abrasion | 0.672 ± 0.204 | 3.29 | p = 0.003* |
|  | Predator attack | −0.687 ± 0.238 | −2.89 | p = 0.008* |
|  | Uninjured conspecifics | 0.584 ± 0.205 | 2.85 | p = 0.009* |
| Experiment 3  (GLM, quasibinomial) | Intercept | −1.086 ± 0.150 | −7.26 | p < 0.001* |
|  | Non-preyed conspecifics | 0.621 ± 0.201 | 3.10 | p = 0.006* |
|  | Preyed conspecifics | 0.498 ± 0.202 | 2.47 | p = 0.024* |
| Experiment 4  (GLM, binomial) | Intercept | −0.952 ± 0.109 | −8.74 | p < 0.001* |
|  | Predator fed jellyfish polyps | −0.675 ± 0.171 | −3.95 | p < 0.001* |
|  | Predator fed sea anemones | −0.405 ± 0.163 | −2.49 | p = 0.013* |

**Table S3.** Pairwise comparisons of estimated marginal means for settlement across treatments. Odds ratios are calculated with the higher settlement odds treatment listed first. Values reported in the Results section may appear as reciprocal odds ratios due to the direction of comparison (e.g., Control vs Predator odour: OR = 0.56 in the Results, compared with OR = 1.77 in this table). Asterisks (*) indicate statistically significant differences (p < 0.05).

| **Experiment** | **Comparison** | **Odds Ratio** | **p-value** |
| --- | --- | --- | --- |
| Experiment 1 | Control / Predator odour | 1.77 | p = 0.041* |
|  | Control / Predator presence | 3.68 | p < 0.001* |
|  | Predator odour / Predator presence | 2.08 | p = 0.037* |
| Experiment 2 | Mechanical abrasion / Predator attack | 3.89 | p < 0.001* |
|  | Uninjured conspecifics / Predator attack | 3.56 | p < 0.001* |
|  | Control / Predator attack | 1.99 | p = 0.020* |
|  | Mechanical abrasion / Control | 1.96 | p = 0.006* |
|  | Uninjured conspecifics / Control | 1.79 | p = 0.023* |
|  | Mechanical abrasion / Uninjured conspecifics | 1.09 | p = 0.969 |
| Experiment 3 | Non-preyed conspecifics / Control | 1.86 | p = 0.006* |
|  | Preyed conspecifics / Control | 1.65 | p = 0.036* |
|  | Non-preyed conspecifics / Preyed conspecifics | 1.13 | p = 0.796 |
| Experiment 4 | Control / Predator fed jellyfish polyps | 1.96 | p = < 0.001* |
|  | Control / Predator fed sea anemones | 1.50 | p = 0.034* |
|  | Predator fed sea anemones / Predator fed jellyfish polyps | 0.76 | p = 0.296 |
